# Experimental Gastric Carcinogenesis in *Cebus apella* Nonhuman Primates

**DOI:** 10.1371/journal.pone.0021988

**Published:** 2011-07-21

**Authors:** Joana de Fátima Ferreira Borges da Costa, Mariana Ferreira Leal, Tanielly Cristina Raiol Silva, Edilson Ferreira Andrade Junior, Alexandre Pingarilho Rezende, José Augusto Pereira Carneiro Muniz, Antonio Carlos Cunha Lacreta Junior, Paulo Pimentel Assumpção, Danielle Queiroz Calcagno, Samia Demachki, Silvia Helena Barem Rabenhorst, Marília de Arruda Cardoso Smith, Rommel Rodriguez Burbano

**Affiliations:** 1 Laboratório de Citogenética Humana, Instituto de Ciências Biológicas, Universidade Federal do Pará, Belém, Brazil; 2 Disciplina de Genética, Departamento de Morfologia e Genética, Universidade Federal de São Paulo, São Paulo, Brazil; 3 Centro Nacional de Primatas, Ministério da Saúde, Ananindeua, Brazil; 4 Departamento de Medicina Veterinária, Universidade Federal de Lavras, Lavras, Brazil; 5 Hospital Universitário João de Barros Barreto, Universidade Federal do Pará, Belém, Brazil; 6 Laboratório de Genética Molecular, Departamento de Patologia e Medicina Forense, Escola de Medicina, Universidade Federal do Ceará, Fortaleza, Brazil; University of Georgia, United States of America

## Abstract

The evolution of gastric carcinogenesis remains largely unknown. We established two gastric carcinogenesis models in New-World nonhuman primates. In the first model, ACP03 gastric cancer cell line was inoculated in 18 animals. In the second model, we treated 6 animals with N-methyl-nitrosourea (MNU). Animals with gastric cancer were also treated with Canova immunomodulator. Clinical, hematologic, and biochemical, including C-reactive protein, folic acid, and homocysteine, analyses were performed in this study. MYC expression and copy number was also evaluated. We observed that all animals inoculated with ACP03 developed gastric cancer on the 9^th^ day though on the 14^th^ day presented total tumor remission. In the second model, all animals developed pre-neoplastic lesions and five died of drug intoxication before the development of cancer. The last surviving MNU-treated animal developed intestinal-type gastric adenocarcinoma observed by endoscopy on the 940^th^ day. The level of C-reactive protein level and homocysteine concentration increased while the level of folic acid decreased with the presence of tumors in ACP03-inoculated animals and MNU treatment. ACP03 inoculation also led to anemia and leukocytosis. The hematologic and biochemical results corroborate those observed in patients with gastric cancer, supporting that our in vivo models are potentially useful to study this neoplasia. In cell line inoculated animals, we detected MYC immunoreactivity, mRNA overexpression, and amplification, as previously observed in vitro. In MNU-treated animals, mRNA expression and *MYC* copy number increased during the sequential steps of intestinal-type gastric carcinogenesis and immunoreactivity was only observed in intestinal metaplasia and gastric cancer. Thus, *MYC* deregulation supports the gastric carcinogenesis process. Canova immunomodulator restored several hematologic measurements and therefore, can be applied during/after chemotherapy to increase the tolerability and duration of anticancer treatments.

## Introduction

Gastric cancer is the fourth most frequent cancer type and the second highest cause of cancer mortality worldwide. Gastric cancer prevalence is influenced by geographic, ethnic, and cultural factors [1]. In addition, adenocarcinoma is the most common digestive tract neoplasia [2].

Nonhuman primates offer a useful model for carcinogenesis studies. Nonhuman primates present close phylogenic relationship to humans and greater similarities with regard to anatomy, physiology, biochemistry, and organ systems, as compared to rodents. They also present a relatively large organ size which enables repeated diagnostic procedures, such as endoscopic examination, blood sample collection and biopsy, on the same animal over a long period of time [3]. Although nonhuman primate models are not common and are expensive compared to rodent models, the long life span observed in nonhuman primates allows for long-term carcinogenic studies.

Chemical carcinogens cause genetic and epigenetic changes that lead to neoplastic transformation. N-methyl-nitrosourea (MNU) is a well-known direct carcinogen, which does not need metabolic activation to exert carcinogenicity. MNU leads to the production of O^6^-methylguanine adducts, resulting in premutagenic lesions and DNA strand breaks. MNU is a nitrosation product of creatinine metabolism that is formed in the presence of nitrites in the acidic gastric environment. MNU production is associated with the ingestion of meat products, cured meats, and seafood [4]. Moreover, it is possible that many species, including humans, are exposed to carcinogenic MNU, generated in their alimentary tract [5]. Thus, tumorigenesis induced by MNU is an interesting model to study gastric cancer.

Canova may be a potential anticancer treatment in patients with gastric carcinoma. It is a complex homeopathic immunomodulator indicated for patients whose immune system is depressed. Canova activates macrophages both *in vivo* and *in vitro* and indirectly induces lymphocyte proliferation [6]. Since innate and adaptive immune responses play a role in tumor surveillance and clearance [7], enhancing the ability to trigger a specific immunologic response against malignant cells is an important anticancer approach.

In the present study, we aimed to establish a gastric carcinogenesis model in *Cebus apella*, a nonhuman primate. We induced stomach tumors by gastric cancer cell line inoculation as well as MNU treatment for the duration of approximately 2.5 years. We evaluated body weight, serum biochemistry values and hematological parameters, as well as MYC proto-oncogene expression and copy number, in these *in vivo* models. In these models, we also assessed if Canova immunomodulator through the enhancement of immunity can contribute to a reduction in adverse effects of anticancer treatment.

## Methods

### 2.1 Nonhuman Primates

36 adult *Cebus apella* (6–7 years old) were evaluated (2.7–3.6 kg). Animals were identified with microchips and were individually housed in Centro Nacional de Primatas, Pará State, Brazil. The animals were fed a healthy balanced diet not enriched with sodium chloride and were weighed daily. In this study, the details of animal welfare and steps taken to ameliorate suffering were in accordance with the recommendations of the Weatherall report, “The use of non-human primates in research”. This study was approved by the Ethics Committee of Universidade Federal do Pará (PARECER MED002-10).

According to a basic veterinary examination, all animals were considered healthy at the time of first blood sampling, endoscopy, and ultrasound. This was confirmed by the animals' behavior as judged by the veterinary check.

### 2.2 Experimental Design

36 animals were randomly separated in six groups and included in 2 studied models:

#### 1^o^ model: cell line inoculation

Negative Control (NC): 6 control *C. apellas* that received saline solution injections instead of Canova or cell line inoculation.

Canova group (CA): 6 *C. apellas* treated with 7 µl/g of Canova during 14 days. These animals did not receive cell line inoculation.
Cell line group (CL): 6 *C. apellas* inoculated with gastric cancer cell line and that received saline solution injections instead of Canova
Cell line plus Canova during 10 days (CLCA1): 6 *C. apellas* inoculated with gastric cancer cell line and after 5 days were treated with 7 µl/g of Canova during 10 days.
Cell line plus Canova during 14 days (CLCA2): 6 *C. apellas* that received gastric cancer cell line inoculation and 7 µl/g of Canova during 14 days (since day 0).

#### 2^o^ model: MNU treatment

6 *C. apellas* treated with MNU. After tumorigenesis, one animal received Canova treatment (MNU group).

### 2.3 Cell line inoculation

One week before the cell line inoculation, the *C. apellas* of CL, CLCA1 and CLCA2 groups were immunosuppressed by a single dose of 50 mg/kg of cyclophosphamide.

Four gastric cancer cell lines were tested: ACP02, ACP03, AGP01 and PG100. The first 3 cell lines were established by our research group from tumor samples of individuals from Northern Brazil [8]. The PG100 a cell line established from a primary gastric adenocarcinoma was obtained from Rio de Janeiro Cell Bank, Brazil (BCRJ). Only the ACP03 cell line, that was establish from an intestinal-type gastric cancer, was inoculated in the animals included in the present study, since it was the only one that was able to start a tumorigenesis process in *C. apella*.

One week after immunosupression, animals of CL, CLCA1 and CLCA2 groups received percutaneous inoculation of 10^10^ cells of ACP03 at the 85^th^ passage between the mucosal and submucosal layers of antral stomach region. Ultrasonography was used to visualize the stomach tissues during cell line inoculation.

### 2.4 Canova treatment

Canova is standardized and authorized by competent agencies for medicinal application. Experiments were performed with commercial Canova donated by ‘Canova do Brasil’, a Brazilian company, which holds the international patent of this medicine (www.canovadobrasil.com.br).

Animals of CA, CLCA1 and CLCA2 groups, as well as one animal of MNU group, were treated with Canova. These animals received 7 µl/g of Canova daily. Canova concentration was determined according the study of Sato et al. in mice model [9]. Dose distributions were calculated at the time of treatment. Canova solution was succussed before treatment and injected by slow infusion in the right femoral vein of *C. apella* in a single dose.

### 2.5 MNU treatment

The animals of the second model received oral fresh doses of MNU (N1517 Sigma-Aldrich, USA) daily for 940 days at a dosage of 16 mg/kg body weight. The animals also received drink water containing MNU in light-shielded bottles daily. Water was restricted during MNU treatment and given *ad libitum* during Canova treatment.

### 2.6 Animal evaluation

Blood samples of all animals were collected for the determination of hematimetric and leukocytic parameters, evaluation of hepatic and renal functions, and serum measurement of C-reactive protein (CRP), folic acid, and homocysteine on day 0 (baseline). During the treatment periods, the animals were inspected daily and their clinical symptoms were recorded. Body weight was determined and peripheral blood from the left femoral vein was collected for all serum analyses.

Chemistry analysis included testing of levels of glucose, urea nitrogen, creatinine, total protein, albumin, globulin, total bilirubin, cholesterol, triglyceride, alanine aminotransferase, aspartate aminotransferase, γ-glutamyl transpeptidase, lactate dehydrogenase, creatine kinase, amylase, calcium, inorganic phosphorus, sodium, potassium, and chloride. Clinical hematology included red blood cell count, hemoglobin, hematocrit, platelet count, white blood cell (WBC) and differential (segmented neutrophil, lymphocyte, monocyte, eosinophil and basophile) counts. Methods and reference values for male adult animal were previously described [10].

CRP was measured by turbidimetric assay as previously described by Price et al. [11]. Serum level of folic acid was measured using chemiluminescent microparticle immunoassay (CMIA) (Abbott System, USA). Serum homocysteine levels were measured with high pressure liquid chromatography (HPLC) (Betamed, Agilent 1100 series, Chromosystems Reagent Kit).

For the first studied model, our results focus mainly in the blood analyses of days 0 and 14. The analysis of folic acid and of homocysteine concentration was also presented on the 9^th^ day. For the second carcinogenesis model, our results focus mainly in the blood analyses of the days 0, 90, 120, 300, 940 and 960.

### 2.7 Tissue samples and gastric mucosa examination

Biopsy samples of gastric normal and non-normal (e.g. non-atrophic and atrophic gastritis, metaplasia, neoplasia) gastric mucosa were collected by endoscopy. Lymphadenectomy was performed to collect axillary and inguinal lymphonode samples.

Gastric mucosa alterations and tumor growth was followed by endoscopy examination and ultrasonography. A pachymeter was used to measure the tumor biopsies.

Histologic analysis of gastric mucosa and axillary and inguinal lymphonode biopsies of *Cebus apella* were embedded into paraffin, cut in 5 µm sections, and stained by hematoxylin and eosin.

### 2.8 MYC expression and copy number analyses

The *MYC* proto-oncogene has been described as a key in the gastric carcinogenic process [12] and, thus, it was select to confirm the presence of a gastric carcinogenesis process. For the first studied model, gastric biopsies of tumorfactions observed on the 9^th^ day after cell line inoculation were used to evaluate the MYC expression and copy number, because only fibrotic lesions were observed in the studied animals on the 14^th^ day. For the second studied model, *C. apella* gastric samples at days 0, 90, 120, 300, 940 and 960 were used to evaluate the MYC expression and copy number.

Fluorescent in situ Hybridization (FISH) was performed to determine *MYC* gene copy number according to the protocol of Pinkel et al. [13] with modifications introduced from Calcagno et al. [14], [15]. Cells were hybridized with digoxigenin-labeled probe (ONPON0824, Bioagency Biotechnology, Brazil) for *MYC* gene region (8q24) and nuclei were counterstained with 4′,6-diamidino-2-phenylindole antifade. Positive *MYC* gene signals appeared as red spots in nuclei and were scored using the criteria of Hopman et al. [16].

Quantitative TaqMan Copy Number Variation (CNV) assays (Applied Biosystems, USA) using real-time quantitative PCR (RT-qPCR) were applied as a confirmation to FISH analysis. RT-qPCR was performed using the FAM/MGB-labeled TaqMan probe for *MYC* gene (Hs01764918_cn) and VIC/TAMRA-labeled TaqMan CNV *RNAse P* (#4403326) for the internal control. RT-qPCR reactions were performed in quadruplicate with genomic DNA (gDNA) according to the manufacturer's protocol (Applied Biosystems, USA). A known human gDNA (Promega, USA) was used for calibration.

MYC mRNA expression was evaluated RT-qPCR. First, complementary DNA was synthesized using High-Capacity cDNA Archive kit according to the manufacturer's protocol (Applied Biosystems, Poland). All RT-qPCR reactions were performed in triplicate for both target gene (*MYC* -Hs00153408_m1) and internal control (*GAPDH* - NM_002046.3). Relative quantification (RQ) of the gene expression was calculated according to Livak and Schmittgen [17]. In the present study, the NC group was designated as a calibrator of the first model where as the baseline values (from day 0) of the animals were used to calibrate the second study model.

Immunohistochemical analyses for MYC protein were performed on formalin-fixed, paraffin-embedded sections. Immunohistochemical staining was performed on the paraffin sections according to Calcagno et al. [15] with primary mouse monoclonal antibody against MYC (dilution 1∶50; DBS, USA). Positive protein expression was defined as clear nuclear imunostaining in more than 10% of the cells.

### 2.9 Data Analysis

In the first model (cell line inoculation), we first evaluated the normal distribution of all data using the Shapiro-Wilk normality test to determine subsequent use of appropriate tests for statistical comparison. Data that were not normally distributed were transformed (z-score transformation) for analysis such that they followed a normal distribution. Analysis of variance in body weight, serum biochemistry values, hematological parameters, *MYC* expression, and copy number were performed by univariate General Linear Model (GLM) followed by Bonferroni post-hoc test. The effect size for GLM analyses was based on Eta Squared (η^2^), in which 0.15 and below was determined as a small effect size; 0.16–0.40, medium effect size; and above 0.40, large effect size. Chi-square test was performed to compare MYC immunostaining among groups. In these analyses, the confidence interval was 95% and *p* values less than 0.05 were considered significant.

In the second model (MNU treatment), only non-parametric tests to repeated measures were used due to the small number of samples. The Friedman test followed by Wilcoxon analysis with Bonferroni's adjustment were performed to analysis of variance in body weight, serum biochemistry values, hematological parameters, *MYC* expression, and copy number at days 0 (baseline), 90 and 120. In these statistical analyses, one animal was excluded due to its early death. In these analyses, p<0.016 was considered statistically significant. Also, due to small numbers of animals, the statistical data analysis concerning MNU treatment among days 300–940 and Canova treatment among days 940–960, was not possible and results are presented in a descriptive format.

## Results

### 3.1 First model – cell line inoculation

Before this study, several methods of cell line inoculation were tested including intraperitoneal, subcutaneous, gavage, orthopic implantation and percutaneous (data not shown). The percutaneous inoculation was the only via that resulted in the development of a tumorigenic process. We also previously detected that 10^10^ cells was the cell number needed to increase the total tumorous percentage compared to small number of cells (10^8^ e 10^9^). However, the high number of cells did not increase the total tumorous percentage (data not shown).

**Figure 1:**
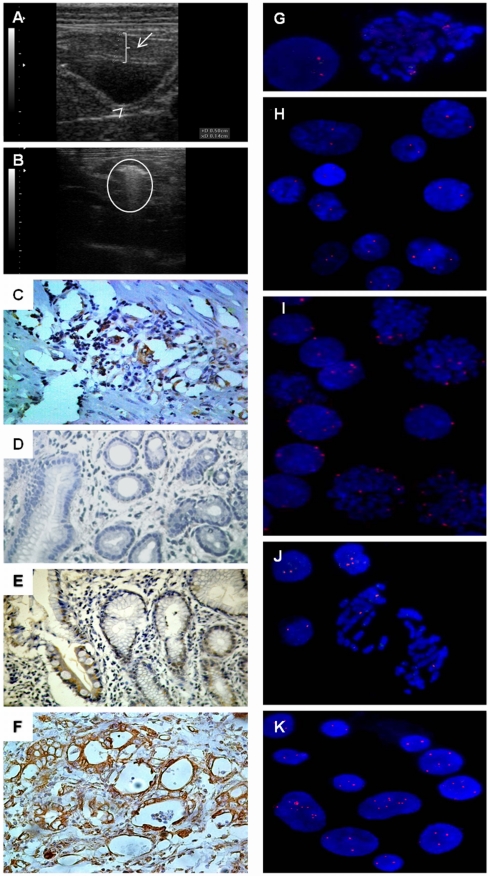
Ultrasonography, immunohistochemistry and FISH analysis in *C. apella* gastric carcinogenesis models. A) ultrasound image showing a “space” between the stomach wall where ACP03 cell line was inoculated and developed a tumor (2.5 cm); B) ultrasound image showing a tumor mass in a MNU-treated animal on the 940^th^ day (5 cm); C); MYC immunoreactivity in a tumor sample of a CLCA1 animal (400×); D); lack of MYC immunoreactivity in non-atrophic gastritis sample in a MNU-treated animal (400×); E) MYC immunoreactivity in intestinal metaplasia sample of a MNU-treated animal (400×); F) MYC immunoreactivity in a tumor sample of a MNU-treated animal (400×); G) lymphocytes of a healthy *C. apella* showing two signals for *MYC* probe (1000×); H) normal gastric mucosa cells of NC animal presenting two *MYC* signals (1000×); I) neoplastic gastric mucosa of CL animal showing *MYC* amplification (1000×); J) intestinal metaplasia sample of MNU-treated animal presenting 1, 2 and 3 *MYC* signals (1000×); K) tumor sample of MNU-treated animal showing *MYC* amplification (1000×). Arrow indicates the space with gastric cancer cell line; arrowhead indicates a normal gastric wall thickness; circle indicates the proliferative process.

In the first model, eighteen *C. apella* received percutaneous inoculation of 10^10^ of ACP03 cells. On the 9^th^ day after cell line inoculation, these animals presented tumorfactions in the antral region of the stomach (Figure 1A). The tumor volumes of CL, CLCA1, and CLCA2 groups were similar and all animals were able to eliminate the tumors. On the 14^th^ day, they presented only an inflammatory zone and fibrotic lesions in the region.

Among the cell inoculation methods, we also evaluated the intraperitoneal inoculation. The intraperitoneal ACP03 inoculation induced lymphatic congestion in *C. apella*. After 48 h of cell line inoculation, the animals presented auxiliary and inguinal lymph node enlargement. Lymphadenectomy was performed and the histopathologic analysis showed only reactive lymphoid hyperplasia.

During this study, the animals in the NC group presented normal levels of the biochemical and hematologic evaluated parameters according to Riviello et al. study in *C. apella* [10]. To our knowledge, there are no CRP, homocysteine, and folic acid reference ranges determined for *C. apella*. However, the non-treated animals presented homocysteine and folic acid levels similar to those described for healthy *Macaca fascicularis* [18].

One week before the cell line inoculation, the *C. apellas* of CL, CLCA1 and CLCA2 were immunosuppressed by a single dose of 50 mg/kg of cyclophosphamide. However, on the day of cell line inoculation, no significant difference was observed among these groups, nor between NC and CA groups, regarding the animal's baseline weight, biochemical, hematologic, folic acid, and homocysteine measurements.

**Figure 2:**
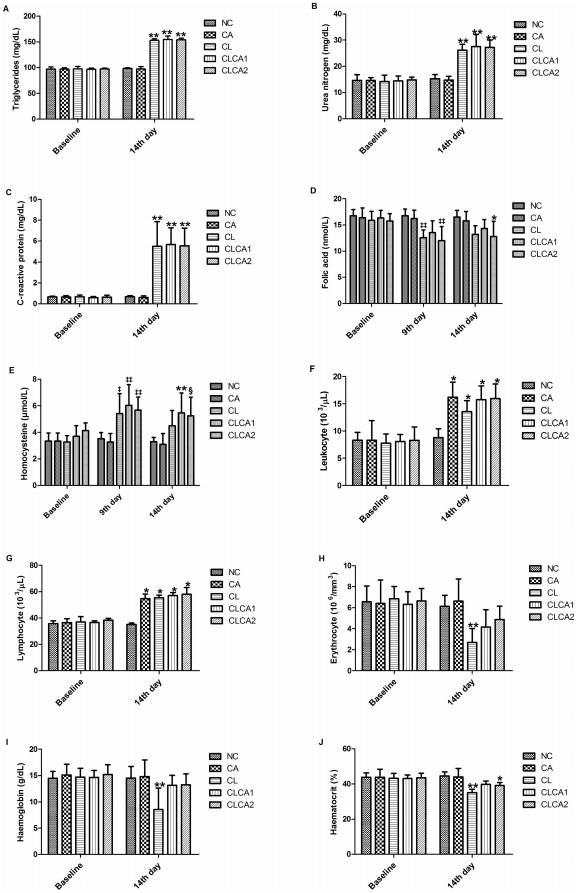
Abnormal biochemical and hematologic measurements in animals of the first carcinogenesis model. A) triglycerides; B) urea nitrogen; C) C-reactive protein; D) leukocyte; E) lymphocyte; F) erythrocyte; G) haemoglobin; H) haematocrit; I) folic acid; J) homocysteine. NC: negative control; CA: Canova group; CL: animals inoculated with ACP03 cell line; CLCA1: *animals* inoculated with ACP03 cell line and treated with Canova during 10 days; CLCA2: *animals* inoculated with ACP03 cell line and treated with Canova during 14 days. N = 6/group. * Significantly different from NC group (p<0.05) on the 14^th^ day. ** Significantly different from NC and CA groups (p<0.05) on the 14^th^ day. ^§^ Significantly different from NC group (p<0.05) on the 14^th^ day. ^‡^ Significantly different from CA group (p<0.05) on the 9^th^ day. ^‡‡^ Significantly different from NC and CA groups (p<0.05) on the 9^th^ day.

Concerning the biochemical analysis, significant changes in triglycerides (F_4,25_ = 335.695, p<0.001, by GLM test; η^2^ = 0.982), urea nitrogen (F_4,25_ = 33.537, p<0.001; η^2^ = 0.843) and CRP (F_4,25_ = 20.135, p<0.001; η^2^ = 0.763) levels were observed among the studied groups on the 14^th^ day (Figure 2A–C, Table S1). The Bonferroni post-hoc analyses demonstrated a significant increase of triglycerides, urea nitrogen, and CRP level in CL, CLCA1 and CLCA2 compared to NC and CA groups (p<0.001, for all pair wise comparisons). No significant difference in biochemical measurements was observed between NC and CA groups. The increase of triglyceride level in the cell-line inoculated animals was inside the normal reference level according to Riviello et al. [10]. On the other hand, abnormal levels of the other biochemical parameter were observed in the cell-line inoculated animals.

To our knowledge, no previous study reported the normal level of CRP in healthy *C. apella*. In the present study, we observed that the range of CRP levels were between 0.34–0.99 mg/dL (n = 36, on day 0). The serum CRP level increased 5.7–13.6 folds due to cell line inoculation.

We observed that the levels of folic acid changed significantly among the studied groups on the 9^th^ (F_4,25_ = 7.446, p<0.001, by GLM test; η^2^ = 0.544) and on the 14^th^ day (F_4,25_ = 4.056, p = 0.011; η^2^ = 0.394). Bonferroni post-hoc analyses demonstrated a significant reduction of folic acid in CL and CLCA2 group than NC (p = 0.008 and p = 0.003, respectively) and CA (p = 0.03 and p = 0.009, respectively) on the 9^th^ day and in animals from CLCA2 group compared to NC group (p = 0.031) on the 14^th^ day, which suggests an effect of cell line inoculation (Figure 2D, Table S1). In the present study, we observed that the range of folic acid level was 13.29–18.84 nmol/dL in healthy *C. apella* (n = 36, on day 0). However, on the 9^th^ day, 4 animals of CL, 4 of CLCA2 and 1 of CLCA1 presented lower levels of folic acid (less than 13 nmol/L) as well as 3 animals of CL, 3 of CLCA2, and 1 of CLCA1 on the 14^th^ day.

We observed that homocysteine levels changed significantly among the studied groups on the 9^th^ day (F_4,25_ = 7.887, p<0.001, by GLM test; η^2^ = 0.558) and on the 14^th^ day (F_4,25_ = 5.6, p = 0.002; η^2^ = 0.473). On the 9^th^ day, CLCA1 and CLCA2 groups presented higher homocysteine levels than animals from NC (p = 0.007 and p = 0.028, respectively, by Bonferroni analyses) and CA (p = 0.003 and p = 0.011, respectively). The CL group also presented higher homocysteine levels than animals from CA group (p = 0.028). However, on the 14^th^ day, only animals from CLCA1 group presented higher homocysteine levels than animals from NC (p = 0.026) and CLCA1 and CLCA2 groups presented higher levels than CA (p = 0.013 and p = 0.29, respectively) group, which suggests some effects of cell line inoculation and of Canova treatment (Figure 2E, Table S1). In the present study, we observed that the range of homocysteine was between 2.5–5.21 µmol/L in healthy *C. apella* (n = 36, on day 0). In addition, we observed that 2 animals of CL, 4 of CLCA1 and 3 of CLCA2 presented homocysteine levels higher than 5.21 µmol/L on the 9^th^ and 14^th^ days.

Concerning hematologic analyses, we observed a significant alteration in leukocyte (F_4,25_ = 10.506, p<0.001, by GLM test; η^2^ = 0.627), lymphocyte (F_4,25_ = 55.213, p<0.001; η^2^ = 0.898), erythrocyte (F_4,25_ = 6.405, p = 0.001; η^2^ = 0.506), haemoglobin (F_4,25_ = 4.798, p = 0.005; η^2^ = 0.434), and haematocrit (F_4,25_ = 12.028, p<0.001; η^2^ = 0.658) counts among the studied groups on the 14^th^ day (Figure 2F–J, Table S1). The Bonferroni post-hoc analyses demonstrated a significant increase of leukocyte and lymphocyte count in CA (p<0.001 and p<0.001, respectively), CL (p = 0.017 and p<0.001, respectively), CLCA1 (p<0.001 and p<0.001, respectively) and CLCA2 (p<0.001 and p<0.001, respectively) as compared to the NC group, which suggests that Canova and cell line inoculation affect leukocyte and lymphocyte levels. Although CA group presented abnormally high lymphocyte count according to Riviello et al. [10], these animals were clinically healthy.

We also detected that CL group presented a significant reduction of erythrocyte (p = 0.006 and p = 0.001, respectively by Bonferroni analyses) and haemoglobin count (p = 0.011 and p = 0.007, respectively) as compared to NC and CA groups, which suggests a cell line inoculation effect that is improved by Canova treatment. According to Riviello et al. [10], all animals of the CL group were anemic. We also observed a significant reduction of haematocrit count in CL and CLCA2 groups as compared to NC group (p<0.001 and p = 0.022, respectively). The CL group also presented reduced haematocrit count as compared to the CA group (p<0.001). According to Riviello et al. [10], 5 animals of the CL and 1 of the CLCA1 group presented abnormal haematocrit level. The hematocrit count difference among groups again suggests a cell line inoculation effect that is in part improved by Canova treatment.

### 3.2 Second model – MNU treatment

We treated six *C. apella* with MNU for a duration of approximately 2.5 years. All animals developed pre-neoplastic lesions and five died of drug intoxication before the development of gastric cancer. All animals presented non-atrophic gastritis on the 90^th^ day. On the 110^th^ day, one animal died from drug intoxication and the other five animas presented atrophic gastristis on the 120^th^ day. On the 134^th^, 140^th^ and 290^th^ days, three more animals died due to drug intoxication. On the 300^th^ day, the two surviving *C. apella* presented intestinal metaplasia in gastric mucosa. One animal died with symptoms of drug intoxication on the 520^th^ day and the last surviving animal developed intestinal-type adenocarcinoma in the antral region of stomach. This tumor was observed by ultrasonography (Figure 1B) and endoscopy on the 940^th^ day and this finding was confirmed by histopathologic analysis. None of the animals developed any other tumor types.

The deceased animals showed the typical symptoms of intoxication: mydriasis, confusion, sleepiness, giddiness, loss of balance, tremor, hyperthermia, low food consumption, nonspecific gastrointestinal symptoms (diarrhea and vomiting), urinary retention, cutaneous eruptions, and caustic and ulcerative oral lesions. They also presented renal, hepatic and respiratory failure, hypokalemia, chronic cholecystitis and steatosis.

**Figure 3:**
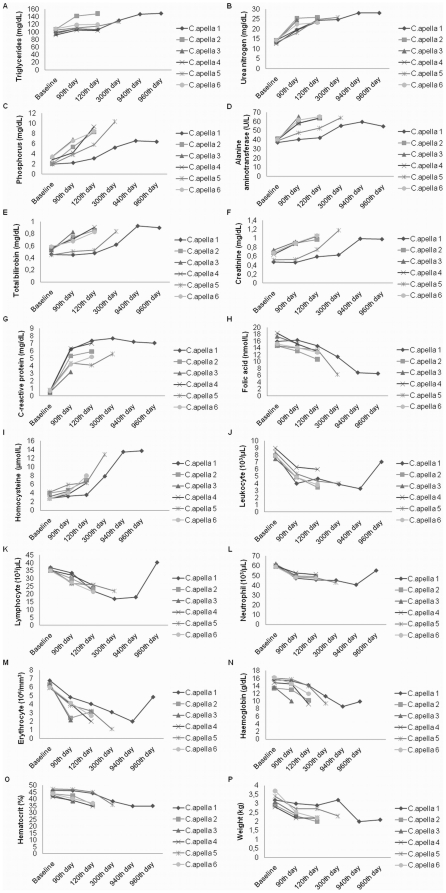
Abnormal biochemical and hematologic measurements in animals MNU-treated and Canova-treated. A) triglycerides; B) urea nitrogen; C) phosphorus; D) alanine aminotransferase; E) total bilirubin; F) creatinine; G) C-reactive protein; H) leukocyte; I) lymphocyte; J) neutrophil; K) erythrocyte; L) haemoglobin; M) haematocrit; N) folic acid; O) homocysteine; P) weight. N = 6 on the 0–90^th^ days (non-atrophic gastritis); N = 5 on the 120^th^ day (atrophic gastritis); N = 2 on the 300^th^ day (intestinal metaplasia); N = 1 on the 940^th^ (gastric cancer development) and 960^th^ (Canova treatment effect).

In the second model of carcinogenesis, we observed that the MNU treatment led to significant changes in triglycerides (χ^2^ = 7.6, df = 2, p = 0.0224, by Friedman test), urea nitrogen (χ^2^ = 10, df = 2, p = 0.0067), phosphorus (χ^2^ = 10, df = 2, p = 0.0067), alanine aminotransferase (χ^2^ = 10, df = 2, p = 0.0067), total bilirubin (χ^2^ = 8.4, df = 2, p = 0.015), creatinine (χ^2^ = 8.4, df = 2, p = 0.015), CRP (χ^2^ = 8.4, df = 2, p = 0.015), folic acid (χ^2^ = 8.4, df = 2, p = 0.015) and homocysteine (χ^2^ = 10, df = 2, p = 0.0067) levels (Figure 3A–I, Table S2). However, no significant difference was confirmed by Wilcoxon test with Bonferroni correction. The largest fold chance was observed in the CRP levels. After 90 days of MNU treatment, the CRP levels increased 5.3–13 folds compared to baseline level. The folic acid concentration was reduced more than 2-fold change and the homocysteine concentration increased almost 5-fold on the 120^th^ day as compared to baseline levels (Figure 3).

Concerning hematologic analyses, we observed a significant alteration of leukocyte (χ^2^ = 8.4, df = 2, p = 0.015), lymphocyte (χ^2^ = 10, df = 2, p = 0.0067), neutrophil (χ^2^ = 10, df = 2, p = 0.0067), erythrocyte (χ^2^ = 8.4, df = 2, p = 0.015), haemoglobin (χ^2^ = 10, df = 2, p = 0.0067) and haematocrit (χ^2^ = 10, df = 2, p = 0.0067) counts during MNU treatment (Figure 3J–O, Table S2). Although no significant difference was confirmed by Wilcoxon test with Bonferroni correction, all animals reached abnormal levels of these hematologic parameters following MNU treatment, consistent with Riviello et al. [10].

The analysis of Canova treatment was based on the observation of its effects in only one animal that developed gastric cancer. After 20 days of Canova treatment, the tumor volume did not change (about 1 cm^3^). In addition, no change (less than 1.2 fold-change) was observed between the 940^th^ and 960^th^ day concerning the biochemical, including folic acid and homocysteine, measurements in the surviving animal. However, we observed that Canova acted mainly on the hematologic measurements. The surviving animal presented more than 2-fold increase in leukocyte, lymphocyte, and erythrocyte counts after Canova treatment as well as a 1.4-fold increase in the neutrophil count. Canova treatment restored normal count of leukocyte, lymphocyte and neutrophil according Riviello et al. [10].

On the 960^th^ day, the survinving *C. apella* was submitted for surgical removal of the tumor. This animal was clinically monitored for one year after the end of the experiment and he did not show any complications resulting from the treatments.

### 3.3 *MYC* copy number

The *MYC* probe for FISH analysis was first tested in the lymphocytes of a healthy *C. apella*. In *C. apella* (Figure 1G), the *MYC* probe had similar efficiency as that observed in our previous results with human cells [19].

Table 1 shows the mean and standard deviation of the *MYC* copy number by FISH and qRT-PCR of the groups included in the first carcinogenesis model. By FISH assay, the number of cells presenting 2 (F_4,25_ = 2578.912, p<0.001, by GLM test; η^2^ = 0.998), 3 (F_4,25_ = 150.51, p<0.001; η^2^ = 0.960), 4 (F_4,25_ = 590.872, p<0.001; η^2^ = 0.99) and 5 or more *MYC* signals (F_4,25_ = 117.013, p<0.001; η^2^ = 0.949) as well as high *MYC* amplification (F_4,25_ = 22.973, p<0.001; η^2^ = 0.786) was significantly different among the studied groups (Figure 1H–I). By qRT-PCR, we also observed that the number of *MYC* copies were significantly different among the studied groups (F_4,25_ = 95.986, p<0.001, by GLM test; η^2^ = 0.939). The Bonferroni post-hoc analyses of FISH results also showed that the number of cells presenting 3, 4, 5 or more MYC signals and high amplification was significantly higher in CL, CLCA1 and CLCA2 groups than in NC and CA groups (p<0.001, for all pair wise comparisons), confirming RT-qPCR results. These *MYC* signal number alterations were observed in the biopsies of all CL, CLCA1 and CLCA2 animals. No significant difference was observed between CL and CLCA groups, suggesting no Canova effect in the *MYC* copy number.

**Table 1:** Immunohistochemistry, relative quantitation of mRNA *MYC* expression and *MYC* gene copy number variation by Taqman and fluorescence *in situ* hybridization in tumor biopsies of animals included in the first carcinogenesis model on the 9^th^ day of treatments.

Group	IHQ	mRNA expression(mean±SD)	CNV(number [mean ± SD])	Nuclei exhibiting *MYC* signals (mean±SD)					
				1 signal	2 signals	3 signals	4 signals	≥5 signals	HA
NC	Negative	-	2 (2.03±0.04)	3.17±1.17	196.83±1.17	-	-	-	-
CA	Negative	−0.51±0.22	2 (2.04±0.56)	3.33±1.21	196.33±1.37	0.33±0.52	-	-	-
CL	Positive^a^	6.45±0.24^b^	5 (4.65±0.43)^a^	5.83±1.94	19.83±5.98^a^	49.17±10.57^a^	69.00±4.29^a^	37.33±6.35^a^	18.33±7.78^a^
CLCA1	Positive^a^	6.24±0.29^b^	5 (4.6±0.34)^a^	3.5±1.76	17.17±6.34^a^	45.33±4.32^a^	70.17±6.911^a^	39.17±7.68^a^	24.67±6.4^a^
CLCA2	Positive^a^	6.01±0.35^b^	4 (4.24±0.52)^a^	4.33±2.34	17.67±5.65^a^	47.5±1.64^a^	72.67±3.14^a^	38.83±3.82^a^	19±8.69^a^

IHC: immunohistochemistry; CNV: copy number variation; HA: high amplification; NC: negative control; CA: Canova group; CL: animals inoculated with ACP03 cell line; CLCA1: *animals* inoculated with ACP03 cell line and treated with Canova during 5 days; CLCA2: *animals* inoculated with ACP03 cell line and treated with Canova during 9 days.

a Significantly different from NC and CA groups (p<0.05).

b Significantly different from CA group (p<0.05).

Table 2 shows the median and interquartile range of *MYC* copy number by FISH and qRT-PCR in samples from animals treated with MNU (second model of carcinogenesis). Using the FISH and RT-qPCR assays, no significant difference was observed among biopsies of day 0, 90 and 120 after Wilcoxon test with Bonferroni correction. On the 300^th^ day, intestinal metaplasia was observed in the two surviving animals. They had approximately 30% of cells with 3 *MYC* signals and about 9.5% of cells with 4 *MYC* signals as determined by FISH and almost 3 copies by qRT-PCR (Figure 1J). The animal that survived the end of the MNU treatment showed a continuous increase in the number of cells with *MYC* amplification during gastric carcinogenesis. On the 940^th^ day, the animal developed intestinal-type gastric cancer and had 46% of cells with 3 or more *MYC* copies, including 5% of cells with high amplification as determined by FISH and 3 *MYC* copies by qRT-PCR (Figure 1K). In the second carcinogenesis model, Canova treatment during 20 days did not appear to change the number of *MYC* copies.

**Table 2:** Immunohistochemistry, relative quantitation of mRNA *MYC* expression and *MYC* gene copy number variation by Taqman and fluorescence *in situ* hybridization in biopsies of MNU-treated animals.

Treatment	IHQ	mRNA expression(median ± interquartile range)	CNV(number [median ± interquartile range])	Nuclei exhibiting *MYC* signals (median ± interquartile range)					
				1 signal	2 signals	3 signals	4 signals	≥5 signals	HA
Baseline	Negative	-	2 (2.04±0.32)	3±1.5	196±3	1±1	-	-	-
MNU/90^th^ day	Negative	1.52±1.06	2 (1.87±0.55)	3±1.5	194±3	2.5±1.75	-	-	-
MNU/120^th^ day^a^	Negative	2.24±1.73	2 (1.92±0.74)	4±0.5	188±5	5±4	2±2	-	-
MNU/300^th^ day^b^	Positive	3.35±0.09	3 (2.85±0.11)	3.5±0.5	115.5±0.5	59.5±1.5	19±3	2.5±2.5	-
MNU/940^th^ day^c^	Positive	4.75	3 (3.12)	3	105	61	16	5	10
Canova/960^th^ day^c^	Positive	5.04	3 (3.04)	4	109	52	18	9	8

IHC: immunohistochemistry; CNV: copy number variation; HA: high amplification.

a Five animals;

b Two animals;

c One animal.

### 3.4 *MYC* expression

Table 1 shows the mean and standard deviation of *MYC* mRNA expression and its immunoreactivity in biopsy samples of animals of the first carcinogenesis model. All normal gastric samples (NC and CA groups) showed standard staining for MYC protein expression. We also observed an association between MYC immunoreactivity and cell line inoculation (χ^2^ = 12, df = 1, p = 0.001). All the tumor biopsies of animals inoculated with ACP03 (CL, CLCA1 and CLCA2 groups) presented MYC protein overexpression (Figure 1C). We did not observe an increase in *MYC* mRNA expression in gastric mucosa of CA group compared to NC group. The *MYC* expression differed among CA, CL, CLCA1 and CLCA2 groups (F_3,20_ = 885.646, p<0.001, by GLM test; η^2^ = 0.993). CL, CLCA1 and CLCA2 groups presented a higher *MYC* expression than the CA group (p<0.001). A six-fold increase in mRNA expression was observed in tumor samples of CL, CLCA1 and CLCA2 groups relative to the NC group. No difference in MYC expression was observed among CL, CLCA1 and CLCA2, which suggests Canova does not effect MYC expression.

Table 2 shows the median and interquartile range of *MYC* mRNA expression and its immunoreactivity in samples from animals treated with MNU (second model of carcinogenesis). By qRT-PCR, we observed a continuous increase in *MYC* expression during MNU-induced gastric carcinogenesis. Although negative MYC immunoreactivity was observed (Figure 1D), the mRNA expression was about 2-fold higher on 120^th^ day (atrophic gastritis) compared to baseline. On the 300^th^ day, we observed an about 3-fold increase of *MYC* mRNA expression in the metaplasia lesions of the two surviving animals in this period relatively to their baseline level. On the 940^th^ day, the surviving animal presented about 5-fold increase in *MYC* mRNA expression relative to its baseline level. MYC nuclear immunoreactivity was observed only in the intestinal metaplasia and gastric cancer biopsies (Figure 1E–F). No change in MYC expression was observed in the tumor biopsy of this animal after 20 days of Canova treatment.

## Discussion

### 4.1 *C. apella* gastric carcinogenesis

Spontaneous tumors have been reported in nonhuman primates, usually due to the aging process [3]. Nonhuman primates offer a useful model for cancer research and other basic research into genetic and immunopathogenesis mechanisms as well as for the development and validation of new therapies for several diseases. *Cebus apella*, a New World monkey, is a convenient model for biomedical studies because they can be easily housed in Primate Research Centers due to their flexibility, opportunism, adaptability, and small size. To our knowledge, this is the first study that established a gastric carcinogenesis model in *Cebus apella*.

Gastric adenocarcinoma is divided mainly into intestinal and diffuse types according to Laurén classification [20]. The intestinal-type gastric cancer progresses through a number of sequential steps beginning with atrophic gastritis followed by intestinal metaplasia, intraepithelial neoplasia, and carcinoma [21]. On the other hand, diffuse-type gastric cancer generally does not evolve from precancerous lesions [2]. Here, we induced intestinal-type gastric adenocarcinoma in *C. apella* by treatment with MNU carcinogen and by human cancer cell line (ACP03) inoculation.

Cell lines derived from human cancers are useful to understand the chromosomal alterations and other molecular alterations in the carcinogenesis process. Cell lines are also an important tool for the study of anticancer treatments in *in vitro* and in animal xenograft models. For the induction of gastric cancer by cell line inoculation in *C. apella*, we initially tested four different gastric cancer cell lines. ACP02, AGP01 and PG100 cell lines did not induce tumors in *C. apella* despite the method of inoculation. Only the ACP03 cell line was able to induce gastric cancer in the animals. The tumorigenic potential of a cell line is attributed to the presence of a subset of cells called cancer stem cells, which have capability to recapitulate the development of the original tumors *in vivo*. The study of human cancer stem cells largely relies on models of xenograft transplantation into immunodeficient mice [22]. Probably, only ACP03 cell line has cancer stem cell proprieties.

One week before ACP03 inoculation, animals of CL, CLCA1 and CLCA2 groups received a single dose of 50 mg/kg of cyclophosphamide. Our group previously observed that white blood cell count decrease after 4 days of cyclophosphamide treatment (unpublished observation). However, total tumor remission was observed after 14 days of ACP03 inoculation probably due to immune system activation. We did not give a second cyclophosphamide dose, because in a previous study we observed that it was lethal to 50% of *C. apella* (unpublished observation). Furthermore, the ACP03 cell line may have low tumorigenic potential and therefore, did not present the same proliferation and metastatic patterns of the original human tumor in these animals. This model can be applied to other gastric cancer cell lines, especially with high tumorigenic potential, in *C. apellas* after their immunosupression. This may be useful for the identification of hematological and biochemical markers that can help to guide immunotherapy cancer treatments.

The presence of gastric tumor due to ACP03 inoculation was confirmed by *MYC* deregulation. The *MYC* proto-oncogene has been described as a key in the gastric carcinogenic process [12]. Groups of genes involved in cell cycle regulation, metabolism, ribosome biogenesis, protein synthesis, and mitochondrial function are over-represented in the *MYC* target gene network. MYC also consistently represses genes involved in cell growth arrest and cell adhesion and also has a direct role in the control of DNA replication [23]. *MYC* amplification has been observed in gastric cancer cell lines and primary stomach tumors [8], [14], [15], [19], [24], [25], [26], [27], [28], [29], [30].

In the first carcinogenesis model, we observed MYC immunoreactivity, amplification (more than 3 *MYC* copies) and mRNA overexpression in the tumor biopsies. Four *MYC* copies was the most frequent copy number alteration in the tumor biopsies supporting our FISH findings in the ACP03 cells culture at the 85^th^ passage [31].

In the second studied model, we induced gastric carcinogenesis by MNU treatment. MNU accumulation leads to the development of several types of tumors in the digestive tract, i.g. in oral cavity, larynx, pharynx and mainly esophagus and stomach of nonhuman primates [3], [32], [33]. MNU induces pre-neoplastic lesions before the development of intestinal type gastric adenocarcinoma [34], usually in antral stomach region of treated animals [35]. In the present study, all MNU-treated *C. apellas* presented pre-neoplastic lesions: non-atrophic gastritis (6 animals), atrophic gastritis (5 animals) and intestinal metaplasia (2 animals). We also observed the development of intestinal-type gastric adenocarcinoma in the antral stomach region of one animal. MNU induced intestinal-type gastric carcinogenesis in *C. apella*, presented the sequential steps similar to those described for humans [21]. Therefore, this model allows for the study of the evolution of intestinal-type gastric cancer, the identification of genes evolved in the early steps of the carcinogenesis process, and the determination of specific targets of gastric neoplastic transformation.

The multistep process of intestinal-type carcinogenesis was also supported by the detection of an increased of mRNA expression and *MYC* copy number during the sequential steps, which begins with atrophic gastritis and is followed by intestinal metaplasia and carcinoma. FISH assay and CNV analysis by qRT-PCR showed that normal mucosa, non-atrophic gastritis, and atrophic gastritis samples of MNU-treated *C. apellas* presented mainly cells with 2 *MYC* copies. Intestinal metaplasia presented cells with 3 *MYC* copies as a clonal alteration (30% of cells) and this gene amplification was observed in about 40% of cells. In gastric cancer samples induced by MNU, almost 50% of cells presented *MYC* amplification, including 5% of cells with *MYC* high amplification. The findings in *C. apella* gastric cancer corroborate our observations in human gastric carcinogenesis, in which the presence of *MYC* amplification, including high amplification, was detected in all human intestinal-type gastric cancer [14], [15], [25], [26], [27], [28] and a significant increase of *MYC* copy number was seen with the evolution of human carcinogenesis process: normal mucosa, intestinal metaplasia, and gastric cancer [27].

*MYC* immunoreactivity was only observed in the intestinal metaplasia and cancer samples, corroborating our previous study with human samples [27]. MYC protein overexpression was previously detected in intestinal metaplasia and neoplastic tissue from all patients with intestinal type gastric cancer [15], [27], [28]. Immunohistochemistry results demonstrated that clonal *MYC* amplification is necessary to induce its protein immunoreactivity.

To our knowledge, this is the third study to describe gastric adenocarcinoma in experimental model in monkeys. Takayama et al. reported 2 nonhuman primates of the Old Word with gastric adenocarcinoma after 10 years of continuous MNU treatment (10 mg/kg) [3]. The small size of *C. apella*, a New World monkey, may contribute to the faster development of gastric adenocarcinoma compared to the Old-World monkeys treated with MNU. Another previous study in literature reported intraepithelial neoplasia induced by ethyl-nitro-nitrosoguanidine treatment in combination with *H. pylori* infection during 5 years in 3 rhesus monkeys [36]. Moreover, the carcinogenesis model described in this study did not infect the animals with *H. pylori*, as frequently described in Mongolian gerbil gastric carcinogenesis model (for review, see [35]).

### 4.2 Biochemical and hematologic measurements in gastric carcinogenesis models

Chronic inflammation is involved with malignant change in several neoplasias. In both studied models, we observed that CRP levels increased significantly with cell line inoculation and MNU treatment. CRP is a representative marker for inflammatory conditions and it has been reported that the risk of cancer is increased when pre-diagnostic CRP levels are high [37]. Elevated CRP has been associated with progressive disease or an advanced stage and a worse survival rate for gastric cancer patients [38]. Chang et al. suggested that although serum CRP is not a specific biomarker for gastric cancer, it might be a potential prognostic biomarker and a promising therapeutic target for gastric cancer patients [39]. In the first carcinogenesis model, elevated CRP may be due to an inflammatory process in the local of cell line inoculation in addition to the tumor cell proliferation. In the second model, we observed that CRP increased 5.3–13 folds on the 90^th^ day of MNU treatment compared to baseline level. The inflammatory process was confirmed by detection of non-atrophic gastritis in all treated *C. apella*. During MNU treatment, the CRP level continually increased until the presence of intestinal metaplasia was elevated in the surviving animal that developed gastric cancer, confirming that CRP is not a specific gastric cancer biomarker.

In both studied models, we observed a reduction of folic acid associated with gastric carcinogenesis. Folic acid maintains genomic stability by regulating DNA biosynthesis, repair and methylation. It has been reported that folic acid deficiency induces and accelerates carcinogenesis by the induction of DNA strand breaks, chromosomal and genomic instability, uracil misincorporation, and impaired DNA repair [40], [41]. Our results in the experimental model of gastric carcinogenesis showed an inverse correlation between folic acid concentration and the risk of gastric cancer development as reported in several experimental and epidemiologic studies with colorectum, esophagus, stomach, pancreas, lungs, cervix, ovary, neuroblastoma cancers, and leukemia [42].

In the first model, homocysteine concentration increased with the presence of gastric cancer on the 9^th^ day. In the second studied model, we observed a continuous increase in homocysteine concentration with cancer development. Serum homocysteine concentration has been suggested as a tumor marker for monitoring cancer patients during anticancer treatment. Elevated circulating total homocysteine has been observed in cancer patients due to cancer cell proliferation and a decline of the high concentration of homocysteine is observed with the death of cancer cells [43]. Hyperhomocysteinemia may induce oxidative stress and DNA hypomethylation, leading to an increase in the risk of cancer, including gastric cancer [44], which corroborate our findings in both carcinogenesis models. In the present study, we did not observe a significant difference between the CL and NC groups on the 14^th^ day of the first studied model, suggesting that homocysteine concentration began to decrease with the tumor regression. Thus, homocysteine concentration can be used for monitoring the efficiency of anticancer treatment.

Concerning hematologic measurements, cell line inoculation leads to anemia and leukocytosis in the first studied model. Anemia is a common complication in patients with inflammatory diseases of many kinds, including cancer. The mechanisms include cytokine-mediated changes in both the production of and the response to erythropoietin, as well as alterations in iron metabolism and increase in the leukocyte production [45]. The increase of white blood cell counts, in agreement with the elevated CRP levels, may be due to an inflammatory process and may have a role in the response against human malignant cells and the tumor remission after 14 days of cell line inoculation.

In the second carcinogenesis model, we observed that urea nitrogen, phosphorus, alanine aminotransferase, total bilirubin and creatinine levels increased with MNU treatment, which may be associated to typical symptoms of drug intoxication presented by 5 *Cebus apella* at the time before their death. We also observed that leukocyte, lymphocyte, neutrophil, erythrocyte, haemoglobinhemoglobin, and haematocrit were significantly reduced with MNU treatment, in agreement with the MNU effects in a broad spectrum of target organs, including particularly the lympho-hematopoietic system [5].

### 4.3 Canova effect in *C. apella* gastric carcinogenesis

In the present study, we observed that Canova acted mainly in hematopoietic system. In the first studied model, Canova induced an increase in leukocyte and lymphocyte and protected ACP03-inoculated animals to present anemia. Although only one animal was evaluated, the Canova treatment seems to restore the normal counts of leukocyte, lymphocyte, and neutrophil and also induced an increase in erythrocyte count that was abnormal after MNU treatment in the second carcinogenesis model.

Abud et al. described that the number of macrophages increased in cultures of bone marrow cells treated with Canova [46], corroborating previous *in vivo* and *in vitro* studies which showed macrophage activation by Canova treatment [47], [48], [49], [50], [51], [52]. According to Abud et al., Canova-active macrophages induce the production of lymphocytes and erythrocytes [46]. Our group also previously observed that Canova induces macrophage activation and indirectly leads to human lymphocyte proliferation *in vitro* [47]. Therefore, these findings are in agreement with those observed in Canova-treated *C. apella*.

In the first studied model, we observed some combinatory effects of cell line inoculation and Canova treatment in homocysteine level on the 14^th^ day, when we observed total tumor regression. However, no conclusion can be made since the normal reference range of homocysteine in *C. apella* is still unknown.

In the second studied model, the tumor volume did not change after 20 days of Canova treatment. We also did not observe differences in the tumor volume among CL, CLCA1 and CLCA2 groups. In addition, MYC expression and copy number did not change with Canova treatment in both studied models. These findings suggest that Canova treatment did not lead to tumor regression in *C. apella* gastric carcinogenesis. However, Sato et al. reported that sarcoma 180 tumor size was significantly smaller in mice treated with Canova (20 days) compared to untreated animals and that 30% of Canova-treated animals presented total tumor regression [9]. These authors also described that all animals of Canova-treated group survived.

Although we did not observed that Canova has a role in gastric tumor regression, the ability of Canova immunomodulator to increase leukocyte count supports a human therapeutic applications such as restoring the hematopoietic system during/after chemotherapy and, thus, increasing the tolerability and duration of anticancer treatments.

## Supporting Information

Table S1 **Abnormal biochemical and hematologic measurements in animals included in the first carcinogenesis model.**
(DOT)

Table S2 **Abnormal biochemical and hematologic measurements in MNU-treated animals and Canova-treated animal of the second studied model.**
(DOC)
