# Supplementary material for: Experimental Gastric Carcinogenesis in Cebus apella Nonhuman Primates
Source: PLoS One. 2011 Jul 21;6(7):e21988. doi: 10.1371/journal.pone.0021988 (PMC3140998; doi:10.1371/journal.pone.0021988)
Supplement: Table S1 — Abnormal biochemical and hematologic measurements in animals included in the first carcinogenesis model. (DOT) [file pone.0021988.s001.dot]

Table S1. Abnormal biochemical and hematologic measurements in animals included in the first carcinogenesis model.

| **Measurement** | **Time** | **Groups (mean** ± **SD)** | | | | |
| --- | --- | --- | --- | --- | --- | --- |
|  |  | **NC** | **CA** | **CL** | **CLCA1** | **CLCA2** |
| C-reactive protein (mg/dL) | Baseline | 0.66 ± 0.07 | 0.65 ± 0.11 | 0.65 ± 0.18 | 0.59 ± 0.11 | 0.64 ± 0.18 |
|  | 14^th^ day | 0.68 ± 0.09 | 0.61 ± 0.15 | 5.51 ± 2.35 | 5.68 ± 1.58 | 5.56 ± 1.68 |
| Triglycerides (mg/dL) | Baseline | 97.33 ± 3.93 | 97.43 ± 2.26 | 97.60 ± 4.72 | 96.89 ± 2.05 | 97.77 ± 1.43 |
|  | 14^th^ day | 98.46 ± 1.37 | 97.79 ± 4.33 | 152.98 ± 3.02 | 154.82 ± 6.66 | 154.07 ± 3.07 |
| Urea nitrogen (mg/dL) | Baseline | 14.67 ± 2.15 | 14.69 ± 1.04 | 14.20 ± 2.46 | 14.51± 1.84 | 14.80 ± 1.12 |
|  | 14^th^ day | 15.32 ± 1.56 | 14.77 ± 1.47 | 26.20 ± 2.25 | 27.55 ± 4.63 | 27.29 ± 2.73 |
| Folic acid (nmol/L) | Baseline | 16.8 ± 1.16 | 16.41 ± 1.87 | 15.9 ± 1.72 | 16.39 ± 1.43 | 15.79 ± 1.4 |
|  | 9^th^ day | 16.77 ± 1.29 | 16.25 ± 1.6 | 12.59 ± 1.46 | 13.56 ± 2.25 | 12.04 ± 2.68 |
|  | 14^th^ day | 16.5 ± 1.32 | 16.83 ± 1.79 | 13.2 ± 1.67 | 14.36 ± 1.71 | 12.81 ± 2.91 |
| Homocysteine (µmol/L) | Baseline | 3.35 ± 0.61 | 3.35 ± 0.61 | 3.28 ± 0.48 | 3.71 ± 0.8 | 4.14 ± 0.58 |
|  | 9^th^ day | 3.53 ± 0.46 | 3.27 ± 0.65 | 5.42 ± 1.5 | 6.035 ± 1.56 | 5.67 ± 0.98 |
|  | 14^th^ day | 3.3 ± 0.32 | 3.11 ± 0.81 | 4.5 ± 1.16 | 5.47 ± 1.5 | 5.25 ± 1.4 |
| Leukocytes (10^3^/μL) | Baseline | 8.33 ± 1.41 | 8.32 ± 3.62 | 7.77 ± 1.69 | 8.08 ± 1.26 | 8.30 ± 2.46 |
|  | 14^th^ day | 8.82 ± 1.59 | 16.22 ± 2.75 | 13.59 ± 2.00 | 15.75 ± 2.53 | 15.97 ± 2.67 |
| Lymphocytes 10^3^/μL | Baseline | 35.81 ± 2.14 | 36.47 ± 3.11 | 36.94 ± 4.14 | 36.65 ± 1.30 | 38.36 ± 1.37 |
|  | 14^th^ day | 35.18 ± 1.14 | 54.67 ± 3.58 | 55.42 ± 2.04 | 57.18 ± 2.19 | 58.11 ± 5.15 |
| Erythrocytes (10^6^/mm³) | Baseline | 6.58 ± 1.48 | 6.42 ± 2.23 | 6.85 ± 1.17 | 6.33 ± 1.20 | 6.65 ± 1.18 |
|  | 14^th^ day | 6.14 ± 1.03 | 6.64 ± 2.10 | 2.88 ± 1.31 | 4.17 ± 1.64 | 4.88 ± 1.26 |
| Haemoglobin (g/dL) | Baseline | 14.50 ± 1.29 | 15.11 ± 2.07 | 14.74 ± 1.65 | 14.65 ± 1.34 | 15.22 ± 1.85 |
|  | 14^th^ day | 14.37 ± 2.21 | 14.98 ± 3.13 | 8.57 ± 4.08 | 13.17 ± 1.89 | 13.25 ± 2.11 |
| Haematocrit (%) | Baseline | 43.92 ± 2.37 | 43.94 ± 4.45 | 43.34 ± 2.72 | 43.16 ± 1.92 | 43.58 ± 2.59 |
|  | 14^th^ day | 44.63 ± 2.29 | 44.09 ± 4.80 | 35.14 ± 1.88 | 39.86 ± 1.80 | 39.21 ± 1.65 |

NC: negative control; CA: Canova group; CL: animals inoculated with ACP03 cell line; CLCA1: animals inoculated with ACP03 cell line and treated with Canova during 10 days; CLCA2: animals inoculated with ACP03 cell line and treated with Canova during 14 days .
