# Supplementary material for: Experimental Gastric Carcinogenesis in Cebus apella Nonhuman Primates
Source: PLoS One. 2011 Jul 21;6(7):e21988. doi: 10.1371/journal.pone.0021988 (PMC3140998; doi:10.1371/journal.pone.0021988)
Supplement: Table S2 — Abnormal biochemical and hematologic measurements in MNU-treated animals and Canova-treated animal of the second studied model. (DOC) [file pone.0021988.s002.doc]

Table S2. Abnormal biochemical and hematologic measurements in MNU-treated animals and Canova-treated animal of the second studied model.

| **Measurement** | **Days** (**median ± interquartile range)** | | | | |  |
| --- | --- | --- | --- | --- | --- | --- |
|  | **Baseline** | **90^th^ day** | **120^th^ day^a^** | **300^th^ day^b^** | **940^th^ day^c^** | **960^th^ day^c^** |
| C-reactive protein (mg/dL) | 0.66 ± 0.11 | 4.87 ± 1.74 | 5.89 ± 1.79 | 6.62 ± 1.03 | 7.21 | 7.04 |
| Triglycerides (mg/dL) | 102.05 ± 9.66 | 110.41 ± 8.38 | 115.08 ± 13.43 | 128.89 ± 1.57 | 147.14 | 149.21 |
| Urea nitrogen (mg/dL) | 13.9 ± 1.07 | 20.98 ± 4.38 | 24.2 ± 0.91 | 25.29 ± 0.51 | 28.00 | 28.08 |
| Phosphorus (mg/dL) | 2.46 ± 1.25 | 4.8 ± 2.32 | 8.35 ± 2.63 | 7.8 ± 2.55 | 6.60 | 6.43 |
| Alanine aminotransferase (U/L) | 39.81 ± 1.2 | 58.115 ± 10.65 | 63.39 ± 11.48 | 59.69 ± 4.31 | 59.74 | 54.78 |
| Total bilirubin (mg/dL) | 0.54 ± 0.09 | 0.7 ± 0.18 | 0.83 ± 0.33 | 0.73 ± 0.11 | 0.93 | 0.90 |
| Creatinine (mg/dL) | 0.66 ± 0.13 | 0.885 ± 0.27 | 0.97 ± 0.3 | 0.91 ± 0.28 | 0.99 | 0.98 |
| Folic acid (nmol/L) | 15.66 ± 2.27 | 14.58 ± 1.25 | 12.98 ± 0.74 | 8.85 ± 2.61 | 6.78 | 6.49 |
| Homocysteine (µmol/L) | 2.95 ± 0.96 | 4.23 ± 0.86 | 6.37 ± 0.58 | 10.36 ± 2.51 | 13.46 | 13.68 |
| Leukocytes (10^3^/μL) | 7.97 ± 0.41 | 4.89 ± 0.36 | 4.34 ± 0.87 | 3.95 ± 0.05 | 3.29 | 7.03 |
| Lymphocytes (10^3^/μL) | 35.53 ± 0.99 | 28.64 ± 4.29 | 23.57 ± 3.78 | 19.45 ± 2.55 | 18.00 | 40.39 |
| Neutrophil (10^3^/μL) | 60.12 ± 1.56 | 49.36 ± 1.59 | 48 ± 1.87 | 44.16 ± 1.05 | 40.68 | 55.21 |
| Erythrocytes (10^6^/mm³) | 6.17 ± 0.39 | 3.96 ± 1.36 | 3.1 ± 0.56 | 2.1 ± 0.98 | 1.98 | 4.85 |
| Haemoglobin (g/dL) | 15.28 ± 1.9 | 14.56 ± 1.74 | 11.97 ± 3.76 | 10.35 ± 0.94 | 8.57 | 9.88 |
| Haematocrit (%) | 43.84 ± 2.61 | 41.41 ± 5.92 | 36.89 ± 8.3 | 37.03 ± 1.23 | 34.75 | 34.89 |

^a^Five animals; ^b^Two animals; ^c^One animal.
